# Implant Restoration of Edentulous Jaws with 3D Software Planning, Guided Surgery, Immediate Loading, and CAD-CAM Full Arch Frameworks

**DOI:** 10.1155/2013/683423

**Published:** 2013-07-29

**Authors:** Silvio Mario Meloni, Giacomo De Riu, Milena Pisano, Francesco Maria Lolli, Alessandro Deledda, Guglielmo Campus, Antonio Tullio

**Affiliations:** ^1^Maxillofacial Surgery Unit, University Hospital of Sassari, 07100 Sassari, Italy; ^2^Dentistry Unit, University Hospital of Sassari, 07100 Sassari, Italy

## Abstract

*Purpose*. The aim of this study was to analyze the clinical and radiographic outcomes of 23 edentulous jaws treated with 3D software planning, guided surgery, and immediate loading and restored with CAD-CAM full arch frameworks. *Materials and Methods*. This work was designed as a prospective case series clinical study. Twenty patients have been consecutively rehabilitated with an immediately loaded implant supported fixed full prosthesis. A total of 120 fixtures supporting 23 bridges were placed. 117 out of 120 implants were immediately loaded. Outcome measures were implants survival, radiographic marginal bone levels and remodeling, soft tissue parameters, and complications. *Results*. 114 of 117 implants reached a 30 months follow-up, and no patients dropped out from the study. The cumulative survival rate was 97.7%; after 30 months, mean marginal bone level was 1.25 ± 0.31 mm, mean marginal bone remodeling value was 1.08 ± 0.34, mean PPD value was 2.84 ± 0.55 mm, and mean BOP value was 4% ± 2.8%. Only minor prosthetic complications were recorded. *Conclusion*. Within the limitations of this study, it can be concluded that computer-guided surgery and immediate loading seem to represent a viable option for the immediate rehabilitations of completely edentulous jaws with fixed implant supported restorations. This trial is registered with Clinicaltrials.gov NCT01866696.

## 1. Introduction

The standard surgical protocol for guided implant placement comprises a diagnostic step, (clinical and radiographic examination), a planning step, and a surgical step, where the surgeon implements what was planned [1].

The growing interest in minimally invasive surgery, together with the possibility of fitting prostheses with immediate function, has led to the development of software and digital workflows allowing for the planning and manufacturing of a surgical guide and provisional prosthesis that can be inserted immediately after the implant surgery step [2–5].

The typical dental implant surgical approach that was introduced in the early 1980s requires two steps [6] and the use of a removable bridge or denture during the healing period. In the 1990s, [7] it was first shown that implants could be placed and restored in a single visit: this procedure, known as immediate loading, needed a full day of coordinated surgical, restorative, and dental laboratory interaction. Advancements in computerized tomography (CT) scans, [8] coupled with computer-assisted treatment planning [9] and a double CT scan approach [3, 10], allowed for the virtual planning of placement of implants in 3-dimensional (3D) orientation relative to the bone, soft tissue, and final planned prosthesis. In 2002, the concept of software planning and surgically guided techniques combined with immediate loading was clinically introduced in Leuven, Belgium [11]. These early treatments were limited to the edentulous maxilla and required a full-thickness flap. Later, the procedure was refined to include flapless implant placement through virtual planning by producing a stereolithographic surgical template incorporating precision titanium drilling sleeves [4]. By retrofitting specialized implant components (implant replicas, and guided cylinders with pin) into the stereolithographic surgical template, an implant-level model, could be produced and a temporary prosthesis could be fabricated for immediate insertion at implant placement [12].

Furthermore, the use of technologies that merge computerized tomography (CT) X-ray imaging and 3-dimensional (3D) planning software allows the surgeon to digitally plan on the computer, the position, length, and diameter of every implant to be placed and, at the same time, helps to prevent damage to vital structures. 

Several studies seem to validate these concepts, but further prospective studies with medium to long term followup are needed [2–4]. The aim of this prospective case series study was to analyze the clinical and radiographic outcomes of 23 edentulous jaws treated with 3D software planning, guided surgery, and immediate loading and restored with CAD-CAM zirconia and titanium full arch frameworks.

## 2. Materials and Methods

### 2.1. Study Design

This was a prospective case series study in which clinical and radiological data analysis was carried out on consecutively treated patients to be prosthetically restored with fixed full arches prosthesis (patients were treated in private practice, while data were collected and analyzed at the University of Sassari). The investigation was conducted according to the principles embodied in the Helsinki Declaration of 1975 for biomedical research involving human subjects, as revised in 2004, and was approved by the Research Committee of the Department of Surgical, Microsurgical and Medical Science, University of Sassari. At the preliminary visit, all patients were duly informed of the nature of the study. 

### 2.2. Selection Criteria

Patients of any race and gender were included in the study if they were at least 18 years old and in good general health, physically and psychologically able to undergo conventional implant surgery and restorative procedures (ASA-1, ASA-2).

Inclusion criteria were edentulous patients or patients with hopeless teeth in need to be restored with full arches prosthesis. 

Exclusion criteria were presence of systemic diseases, (i.e., haematologic disease, uncontrolled diabetes, serious coagulopathies, and diseases of the immune system); irradiation to the head or neck region within 12 months before surgery; severe bruxism or clenching habits; pregnancy; poor oral hygiene; poor motivation to return for scheduled follow-up visits. The included patients were treated in the same dental office, by surgeons and prosthodontists with considerable clinical expertise in immediate loading procedures.

According to the previously criteria mentioned, a total of 20 patients (23 ridges: 8 mandible, 15 maxilla) have been rehabilitated from April 2006 to January 2009 with an immediately loaded implant supported fixed full prosthesis. Following the 3D software treatment planning, four to six implants (Nobel Replace Tapered Groovy; Nobel Biocare AB, Goteborg, Sweden) were placed according to a guided implant protocol (NobelGuide Nobel Biocare) in the mandible or in the maxilla. A total of 120 fixtures were placed, 22 of which were placed in fresh postextraction sockets.

### 2.3. Clinical Procedure

**Figure 1:**
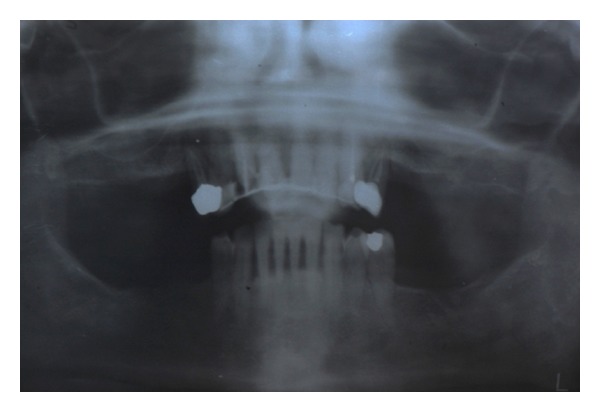
Preoperative panoramic radiograph.

For all cases, the following surgical and prosthetic protocol was followed. The patients were subjected to a clinical evaluation, and a medical history was taken. Informed consent was collected. Preliminary radiographic screening was performed using panoramic orthopantomographs (Figure 1).

A careful clinical examination of the patients was performed assessing jaw sizes and relationships, bone volume, and occlusal relationships. 

 Eligible patients received oral hygiene instructions, and impressions and baseline photographs of their dentition were taken. Registrations with a facial bow were also taken for aesthetic/functional evaluation. After the diagnostic phase, for each patient, the teeth to be removed and the implants to be inserted were determined.

Two different approaches were used:

- guided implant insertion in healed ridges (flapless or with flap when needed to preserve keratinized tissue).
- guided flapless implant insertion with some implants inserted in fresh extraction sockets and others in healed ridges.

From each impression, a wax setup was developed and in case of implant insertion in healed a removable prosthesis ridges, (used as radiographic guide) was customized according to the aesthetic and functional evaluations.

When guided flapless implant insertion in either healed or fresh extraction sites was planned, a teeth-supported provisional prosthesis was customized and only three or four hopeless teeth were left in the oral cavity of each patient to temporarily support the provisional prosthesis, while the other teeth were extracted. After 4–6 months, a radiological template was made according to the aesthetic and functional wax setup, and a silicone interocclusal record was made to be used as a radiographic index.

**Figure 2:**
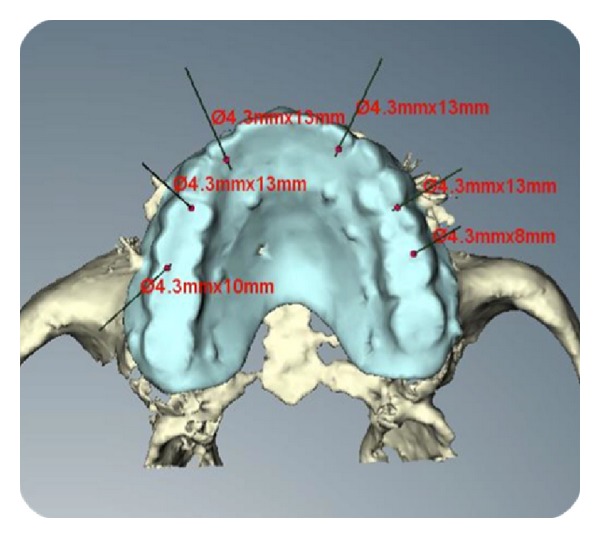
Virtual implant planning. Occlusal view.

In accordance with the NobelGuideTM data acquisition protocol (Nobel Biocare, Gothenburg, Sweden), two CT scans were performed: one with the patient wearing the radiographic guide as well as the radiographic index, the other with the radiological template alone. CT scan data were transferred to the software program for 3D diagnostic analysis and virtual implant planning (Figure 2). Anatomical conditions had to allow the placement of at least four to six implants in the ideal position for prosthetic rehabilitation. When an implant was planned with the tooth in situ, it was easy to see the vestibular and palatal cortical bones. After bone volume analysis, implants were planned on a palatal or lingual aspect and the implant platform virtually positioned at a level of 1.0 mm or less under the coronal part of the vestibular alveolar crest. The software planning data were sent to the manufacturer (Nobel Biocare, Gothenburg Sweden), where a surgical template with hollow metallic sleeves was produced to guide the implants according to the virtually planned position. Based on the surgical guide and the cast model obtained, metal-acrylic resin screw-retained provisional prostheses were prefabricated.

The surgical procedure was performed under local anesthesia with articaine chlorhydrate plus 1 : 100,000 adrenaline (Pierrel S.p.A, Milan, Italy). All patients were given diazepam (Valium, 10 mg, Roche, USA) as a sedative agent before surgery. Antibiotics amoxicillin 875 mg and clavulanic acid 125 mg (GlaxoSmithKline S.p.A., Verona, Italy) were given 1 h before surgery and twice a day for 6 days thereafter. An anti-inflammatory drug (ketoprofen 80 mg Dompé S.p.A, Milan, Italy) was administered twice a day for 4 days postoperatively. An antacid agent (omeprazole 20 mg, Pensa Pharma S.p.A, Milan, Italy) was administered on the day of surgery and once daily for 6 days postoperatively. Each patient rinsed with chlorhexidine gluconate (0.2%) for 1 min before the intervention (Curasept, Curaden healthcare srl, Saronno, Varese, Italy). 

**Figure 3:**
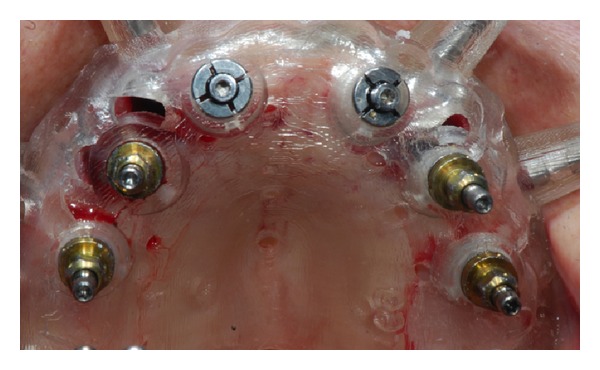
Guided flapless implant insertion in healed sites and fresh extraction sockets.

In cases of implants planned in fresh extraction sockets, teeth used for temporary support of a provisional prosthesis were removed with an atraumatic technique 30 minutes before surgery. Surgical templates were then placed intraorally in the correct position and in relation to the opposing arch (a silicone occlusal index was used) and then fixed with three or more anchor pins. Considerable care was taken when placing the surgical template. After correct placement and stabilization of the surgical template, flapless implant surgery was performed in accordance with the drilling protocol for the type of implant used (Figure 3) (NobelReplace Tapered Groovy, Nobel Biocare, Gothenburg, Sweden) in 16 ridges (15 maxillas, 1 mandible), while in 7 mandibular cases, a small full-thickness flap was raised in order to preserve keratinized tissue. Implants were placed with a preset insertion torque of 35 to 45 Ncm. The implant length ranged from 8 to 13 mm and the implant diameter was 3.5, 4.3, or 5 mm. In the case where full-thickness flaps were raised, flaps were sutured after implant installation with interrupted sutures Vicryl 4.0 sutures (Vicryl, Ethicon J&J International, St-Stevens-Woluwe, Belgium). Sutures were removed after 7 days, when possible, without removing the immediate loaded prosthesis, or left in place until resorbed.

 When fixtures were installed in fresh extraction sockets, the space between the vestibular cortex and the implant surface was always filled with bovine bone grafts (Bio-OSS Geistlich, Wolhusen, Switzerland), and collagen or connective tissue was used to cover the graft and thicken soft tissues.

**Figure 4:**
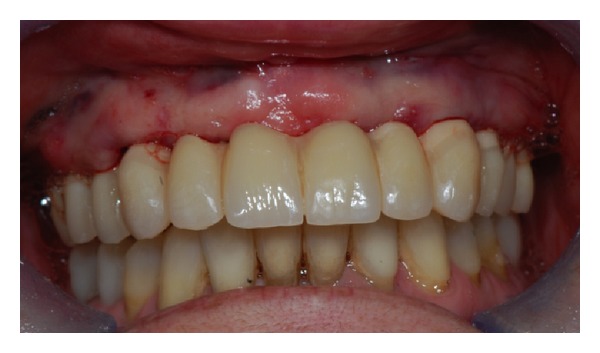
Immediate loading with screw-retained temporary prosthesis.

**Figure 5:**
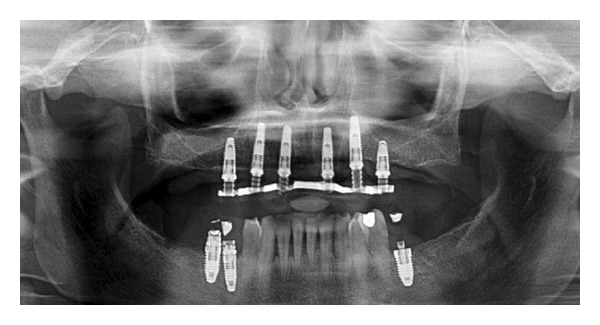
Panoramic radiograph after immediate loading.

117 out of 120 implants were immediately loaded with the prefabricated screw-retained provisional prosthesis (Figures 4 and 5), while three remaining implants placed with an insertion torque less than 35 Ncm were delayed loaded. When needed, minor adjustments of provisional restorations were made to correct occlusion. In all postextraction sites, the profile of the prosthesis was relined with resin to provide better support for the soft tissues and to achieve a passive fit of the temporary abutment. Ice packs were provided and a soft diet was recommended for 1 month. All patients were included in an implant maintenance program. Smokers were asked to refrain from smoking for at least 48 h postoperatively. Chlorhexidine gluconate mouthwash (0.2%) was prescribed for 1 min twice a day for 2 weeks after surgery. The patients were instructed on oral hygiene, and they returned every 3 months for a maintenance appointment. Scheduled visits after surgery were after 1 week, 2 weeks, 1 month, 3 months, and 6 months.

**Figure 6:**
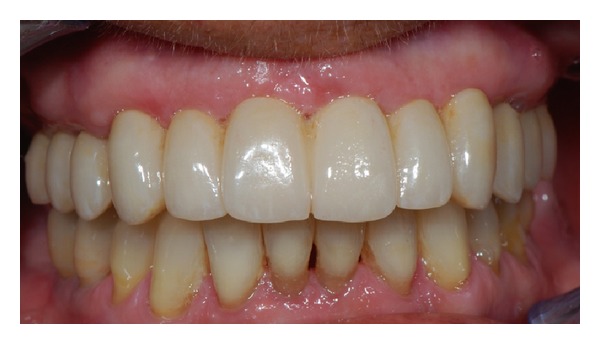
Final restoration with zirconia ceramic screw-retained prosthesis.

**Figure 7:**
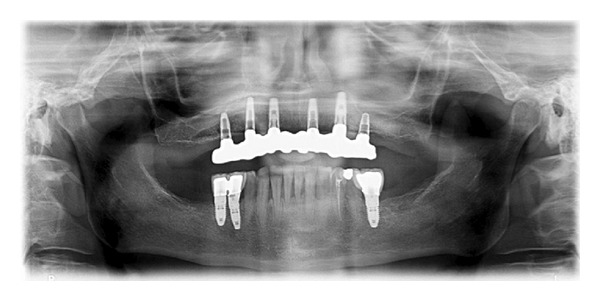
Panoramic radiograph 30 months after loading.

After 6 months, the prostheses were removed and the implants were individually tested for stability. The definitive prosthetic restorations, either Procera implant bridge titanium as the framework and resin as aesthetic material (18 ridges) or Procera implant bridge zirconia ceramic (5 ridges) were then inserted (Figures 6 and 7). After final prosthesis delivery, patients were checked every six months.

The following outcome measures were used.

#### 2.3.1. Success and Failure Criteria

The success criteria to be used in this investigation are a modification of the success criteria suggested by van Steenberghe et al. [4]. The success criteria of the investigation have been determined as follows.

- A “successful implant” is an implant which
  - does not cause allergic, toxic, or gross infectious reactions either locally or systemically;
  - offers anchorage to a functional prosthesis;
  - does not show any signs of fracture or bending;
  - does not show any mobility when individually tested by tapping or rocking with a hand instrument;
  - does not show any signs of radiolucency on an intraoral radiograph using a paralleling technique strictly perpendicular to the implant-bone interface.
- A “surviving implant” is when the implant remains in the jaw and is stable, and when the subject's treatment is functionally successful even though all the individual success criteria are not fulfilled.
- A “successful prosthesis” is a prosthetic reconstruction that is stable and in good function.
- A “failed implant” is an implant that has been removed, fractured beyond repair, or cannot be classified as a successful or surviving implant.

#### 2.3.2. Complications

All types of complications, either mechanical or biological, were recorded.

### 2.4. Marginal Bone Levels and Marginal Bone Remodeling

Peri-implant marginal bone levels were evaluated on intraoral digital radiographs taken with the parallel technique at the time of implant placement, at 6, 12, and 30 months and after loading. If radiographs were inconclusive, they were repeated. A blinded radiologist, unaffiliated with the study center, interpreted all radiographs. The distances from the mesial and distal interproximal bone to the reference point (the horizontal interface between the implant and abutment) were measured with an image software measurement tool (NIH Scion Image Corporation 4.0.2, Frederick, MD, USA) calibrated against the space between two threads to the nearest 0.1 mm, and the mean of these two measurements was calculated for each implant. The measurements were recorded with reference to the implant axis. Mean values and standard deviation were recorded.

The marginal bone remodeling was calculated as the difference between the reading at the examination and the baseline value. Mesial and distal bone height measurements were averaged for each implant. Mean values and standard deviation were recorded.

#### 2.4.1. Peri-Implant Mucosal Response

Probing pocket depth (PPD) in mm and bleeding on probing (BOP) in % were measured by a blinded operator with a periodontal probe (UNC 15) at 6 months, 12 months, and 30 months after loading. Three vestibular and 3 lingual values were collected for every implant by the same dentist. Mean values and standard deviation were recorded.

## 3. Results

Twenty consecutive patients, 8 males and 12 females, with a mean age of 56 (range, 39–78) were treated, and 23 ridges were restored. No patient dropped out of the study, and the followup was at least 30 months after implant insertion for all cases. No deviations from the protocol occurred. Data were collected in sheets (Excel, Microsoft, Redmond, WA USA) at baseline, 6 and 12, 30 months after implant loading. A total of 120 implants were placed, 117 of them with an insertion torque between 35–45 Ncm, and were immediately loaded while three fixtures were delayed loaded.

### 3.1. Implant Survival

Three out of 117 immediately loaded implants and 1 out of 3 delayed loaded implants were lost in 3 patients, 6 months after implant insertion. No other implants failed in the remaining part of the study accounting for a CSR of 97.7% (calculated on 120 inserted implants) after 30 months. Lost implants were replaced. 

### 3.2. Prosthesis Success

No Prosthesis failures were recorded: all final prosthetic reconstructions were stable and in good function after 30 months.

### 3.3. Complications

No major biological complications were recorded. Two patients had peri-implant mucosal inflammation with BOP after 6 months. Improved oral hygiene reduced the peri-implant inflammation. 

No major mechanical complications occurred. Fourprovisional acrylic bridges fractured 2–4 months after immediate loading and were repaired. Two zirconia ceramic implant bridges had ceramic chipping at 10 and 11 months after loading, which were easily repaired. Four resin titanium bridges experienced fracture of the acrylic resin after 10 and 12 months and were repaired by the dental technician.

### 3.4. Peri-Implant Marginal Bone Levels

The average marginal bone level at baseline was 0.16 ± 0.10 (calculated on 117 immediate loaded implants). Mean marginal bone level after 30 months was 1.25 ± 0.31 (calculated on 114 survived immediate loaded implants) (Table 1).

### 3.5. Peri-Implant Marginal Bone Remodeling

The average marginal bone remodeling from baseline to last radiological control (30 months) was 1.08 ± 0.34 (calculated on 114 survived immediate loaded implants) (Table 2).

### 3.6. Peri-Implant Mucosal Response

After 30 months, mean PPD value was 2.84 ± 0.55 mm, and mean BOP value was 4% ± 2.8%. (calculated on 114 survived immediate loaded implants) (Tables 3 and 4).

## 4. Discussion

Correct diagnosis and accurate implant planning are key factors for success in implant rehabilitation. The use of advanced 3D software planning, by using converted CT scans, reduces the risk of damaging nearby vital structures and allows more precise planning than conventional CT scans [13].

According to these concepts, computer-guided minimally invasive implant treatment protocols promise to revolutionize the way implant dentistry is practiced.

In fact, the use of guided surgery reduces the risk of damaging anatomical structures while exploiting the residual bone volume and also allows a sensible reduction of surgery time while delivering immediate implant supported temporary bridges [3, 5, 10].

In this article, all cases used a full arch immediate loading technique with temporary bridge, avoiding a direct definitive bridge delivery. In fact, deviation of the virtual plan of few degrees can prevent a perfect passive fit of the bridge. To overcome this problem for immediate loading, we always use a screw-retained metal-acrylic temporary bridge and a passive fit, it is obtained directly in the oral cavity using resin to connect the temporary cylinder to the metal framework and avoid using a guided abutment. Then after at least 6 months, the final CAD/CAM customized implant bridge is directly connected to the implant neck, and the passive fit of the framework (titanium or zirconia bridge) is clinically evaluated before final prosthesis delivery.

Flapless implant placement delivers several advantages to the patient: minimal swelling, pain, and discomfort, elimination of a second surgical procedure, maintaining the soft tissue architecture, and leaving the periosteum intact on buccal and lingual aspect of the ridge which, in turn, maintains a better blood supply and thus reduces the likelihood of bone resorption [14, 15].

On the other hand, flapless guided surgery presents increased risks compared to open surgical approaches due to the inability of the surgeon to verify the accuracy of the guide intraoperatively and to compare the clinical implant position with the virtual planned position. For this reason, guided flapless surgery requires greater surgical experience in implant placement and, in particular, guided surgery, and it does not represent the first option for young clinician not sufficiently trained in guided implant insertion. In spite of clinical skills in flapless implant insertion, an open flap approach is needed when either only few millimeters of keratinized tissue are present, or when an osteoplasty is required for prosthetic reasons. We think that this approach is mandatory in most of the mandible restorations. 

One of the main advantages of computer-guided technology in implant dentistry is the better control of the implant axis in relation to the prosthetic tooth position. This leads to a higher predictability of the treatment outcome with subsequent better patient information about the aesthetic final result. 

Despite some early skepticism about the usefulness of these techniques, it is well known that guided templates are more precise than conventional surgical guides produced by the laboratory when it comes to the soft tissue contours [2].

Different software planning and guided surgery systems exist with differences between them; NobelGuide protocols differentiate from others for the double CT-scanning approach, which allows for better visualization of soft tissue thickness and uses a calibration procedure which has the benefit of more precise implant installation with better visualization of the correct implant axis. This type of guided surgery has been tested in many clinical studies [2, 5, 16], including extremely challenging situations such as patients treated after oncologic resection or gun shot traumas [17].

Nevertheless, some limitations still exist in the application of this new technology. Minimum bone volumes and attached soft tissue are required to place the implant in ideal position in relation to the planned restoration. These limitations could be present in every kind of implant procedures [18]. In cases of flapless guided implant installation, a large amount of attached gingiva is needed, and major skills are required to evaluate if part of the implant surface is out of the bone and or if the planned implant position does not match perfectly the clinical situation.

Other critical objections include increased costs, investment costs for the software, the training of the dentists, and the stereolithographically produced surgical templates. Investment of time for the planning process also means an increase in costs. However, three-dimensional analysis of computer tomograms and virtual implant placement, once the operator is familiar with the software, are fast and allow one easy and more precise prosthetic guided implant planning in challenging situation such as resorbed ridges or implant installation in fresh extraction sockets, often avoiding bone grafting procedures [19, 20]. Reduced patient morbidity is another important aspect that cannot be calculated directly in money but is beneficial for the patients.

During the last few years, studies have increasingly investigated the clinical and radiological outcome of guided implant placement and they seem to confirm the high predictability of 3D planning software in regards to their ability to offer precision between what is planned and then executed surgically [21]. Careful surgical and prosthetic planning is valuable in order to avoid implant misplacement [22].

Sanna et al. have reported in a 5-year prospective trial a cumulative survival rate of 95% with mean marginal bone changes of 2.6 mm in smokers and 1, 2 in non smokers patients [16]. Malo et al., in 2007, reported a CSR of 97.8% with a mean marginal bone loss of 1.9 after 12 months of followup [5]. Merli et al. in a clinical case series reported similar results [3].

Abboud et al. compared two different stereolithographic surgical guide systems, NobelGuide (Nobel Biocare) and SimPlant (Materialise), obtaining similar results in transferring the planned implant positions to the surgical field and allowing the placement of prefabricated provisional [23]. Platzer et al. obtained similar results in a pilot study [24].

Our team has recently retrospectively investigated NobelGuide protocol in full edentulous maxillae with a followup of 18 months with a CSR of 97.8% [2] and published a pilot study on a modified protocol for implant installation in free-flaps [17]. High survival rate and marginal bone loss comparable with other procedures were reported and higher patient satisfaction in this challenging situation too.

Within the limitations of this study, mainly the relatively low number of patients treated and short observation period, it can be concluded that according with the literature reviews [25], computer-guided surgery and immediate loading seem to represent a viable option for the immediate rehabilitations of completely edentulous jaws with fixed implant supported restorations.

## Figures and Tables

**Table 1:** Peri-implant mean marginal bone levels (*n*: number of implants).

	Mean marginal bone level	
Implant installation	12 months	30 months
*n* = 117	*n* = 114	*n* = 114
0.16 ± 0.10	1.01 ± 0.28	1.25 ± 0.31

Values represent mean ± SD.

**Table 2:** Peri-implant mean marginal bone remodeling (*n*: number of implants).

Mean marginal bone remodeling	
12 months	30 months
*n* = 114	*n* = 114
0.85 ± 0.30	1.08 ± 0.34

Values represent mean ± SD.

**Table 3:** Mean PPD values in mm (*n*: number of implants).

	Mean PPD	
6 months	12 months	30 months
*n* = 114	*n* = 114	*n* = 114
2.77 ± 0.57	2.78 ± 0.65	2.84 ± 0.56

Values represent mean ± SD.

**Table 4:** Mean BoP values in % (*n*: number of implants).

	Mean BOP values	
6 months	12 months	30 months
*n* = 114	*n* = 114	*n* = 114
7.0 ± 3%	6.4 ± 3.4%	4% ± 2.8%

Values represent mean ± SD.
